# Long-term outcome of the humoral and cellular immune response of an H5N1 adjuvanted influenza vaccine in elderly persons: 2-year follow-up of a randomised open-label study

**DOI:** 10.1186/1745-6215-15-419

**Published:** 2014-10-29

**Authors:** Paul Gillard, Didier Giet, Stéphane Heijmans, Mamadou Dramé, Karl Walravens, François Roman

**Affiliations:** GSK Vaccines, Wavre, Belgium; Department of General Medicine, University of Liege, Liège, Belgium; ResearchLink, Linkebeek, Belgium; GSK Vaccines, King of Prussia, PA USA; GSK Vaccines, Rixensart, Belgium

**Keywords:** Avian influenza, H5N1, Pre-pandemic vaccine, Persistence, Elderly population, Cell-mediated immune response

## Abstract

**Background:**

Older individuals often have a reduced immune response to influenza vaccination, which might be improved by administering a higher vaccine dose. We compared the immune response to two single doses of the AS03_A_-adjuvanted H5N1 pandemic vaccine (3.75 μg hemagglutinin of A/Vietnam/1194/2004) with that of two double vaccine doses (7.5 μg hemagglutinin) in adults aged ≥61 years. Here we report the 2-year persistence of the humoral and cellular immune response.

**Methods:**

In this phase II, open-label study, healthy participants aged 61 to 88 years (median 68 years) were randomised (3:1:3:1) to receive two single doses of the AS03_A_-adjuvanted vaccine (1xH5N1-AS) or the non-adjuvanted vaccine (1xH5N1), or two double doses of the AS03_A_-adjuvanted vaccine (2xH5N1-AS) or the non-adjuvanted vaccine (2xH5N1), 21 days apart. Serum haemagglutination inhibition antibodies and cellular immune responses against A/Vietnam/1194/2004 were measured in all groups at months 12 and 24; neutralising antibodies were assessed in a subset of the adjuvanted groups. Serious adverse events and adverse events of specific interest were recorded.

**Results:**

At month 24, haemagglutination inhibition antibody seroprotection rates were 37.2% (95% CI 27.0% to 48.3%) for 1xH5N1-AS, 30.9% (95% CI 21.1% to 42.1%) for 2xH5N1-AS, 16.2% (95% CI 6.2% to 32.0%) for 1xH5N1, and 8.3% (95% CI 1.0% to 27.0%) for 2xH5N1. Haemagglutination inhibition antibody geometric mean titres were 17.6 (95% CI 13.7 to 22.5) for 1xH5N1-AS, 18.4 (95% CI 14.2 to 23.8) for 2xH5N1-AS, 12.3 (95% CI 8.9 to 16.9) for 1xH5N1 and 9.8 (95% CI 6.7 to 14.4) for 2xH5N1. The median frequency of antigen-specific CD4^+^ T cells per 10^6^ T cells (25th quartile; 75th quartile) was 852 (482; 1477) for 1xH5N1-AS, 1147 (662; 1698) for 2xH5N1-AS, 556 (343; 749) for 1x-H5N1 and 673 (465; 1497) for 2xH5N1. Neutralising antibody geometric mean titres were 391.0 (95% CI 295.5 to 517.5) in the 1xH5N1-AS group and 382.8 (95% CI 317.4 to 461.6) in the 2xH5N1-AS group.

**Conclusions:**

Antibody levels declined substantially in all groups. Seroprotection rates, geometric mean titres for haemagglutination inhibition antibodies, and CD4^+^ T-cell responses tended to be higher in the AS03_A_-adjuvanted groups. There was no clear benefit, in terms of long-term persistence of the immune response, of doubling the dose of the adjuvanted vaccine. No safety concern was observed up to 24 months post-primary vaccination.

**Trial registration:**

NCT00397215 (7 November 2006).

**Electronic supplementary material:**

The online version of this article (doi:10.1186/1745-6215-15-419) contains supplementary material, which is available to authorized users.

## Background

Outbreaks of avian influenza H5N1 viruses emerged in 1997 and remain a considerable threat. A key strategy for pandemic preparedness involves the development of a pre-pandemic vaccine to prime the immune system against H5 variants [1]. Partial cross-protection may have a considerable impact on infection rates during early pandemic stages [2, 3]. The induction of long-lasting immune memory by a pre-pandemic vaccine may allow for sufficient protection to be induced by just a single booster vaccine [1, 4, 5].

An H5N1 pre-pandemic AS03_A_-adjuvanted vaccine based on the A/Vietnam/1194/2004 clade 1 strain is licensed for prophylaxis of influenza in an officially declared pandemic situation, with a schedule of two injections administered 3 weeks apart, from the age of 18 years onwards [6]. This vaccine has been shown to induce a cross-reactive immune response against drifted clade 2 H5N1 strains [7–11].

In adults aged 18 to 60 years, a formulation of this adjuvanted H5N1 vaccine containing 3.75 μg hemagglutinin was sufficient to meet all US Center for Biologics Evaluation and Research and European Committee for Human Medicinal Products (CHMP) immunologic licensure criteria [7]. Older persons often experience a reduced immune response to influenza vaccination, which is thought to be, at least partially, due to immunosenescence [12]. It is therefore anticipated that these individuals may need higher doses to enhance their immune response after vaccination.

We previously reported on the immunogenicity and safety of a single (3.75 μg) or double (7.50 μg) dose of the AS03_A_-adjuvanted H5N1 pandemic vaccine in individuals aged ≥61 years in a randomised, open-label study (NCT00397215) conducted in Belgium and Italy [13]. Both the single and the double dose regimens were found to be well tolerated and highly immunogenic when administered as a two-dose schedule 21 days apart. Although a double dose elicited a stronger immune response, the single dose was sufficient to meet all CHMP criteria for influenza vaccines in the elderly population. The immune response to both the single and double adjuvanted dose remained high after 6 months [13].

The current manuscript presents follow-up data from the population in Belgium on the persistence of the humoral and cellular immune responses up to 2 years after the first vaccination with the H5N1 vaccine. We assessed the persistence of the immune response against the vaccine-homologous A/Vietnam/1194/2004 strain, as well as against the heterologous A/Indonesia/05/2005 strain. Safety data, in terms of serious adverse events (SAE) and adverse events of specific interest (AESI) up to 24 months after the first vaccination, are also presented.

## Methods

### Study vaccines

The H5N1 inactivated, split-virion recombinant vaccine was manufactured by GSK Vaccines (Dresden, Germany), as described elsewhere [7, 8]. The vaccine contained 3.75 μg hemagglutinin of the A/Vietnam/1194/2004-like NIBRG-14 clade 1 strain (National Institute for Biological Standards and Control). The adjuvanted vaccine (*Prepandrix*™, GSK Vaccines) contained AS03_A_, an oil-in-water emulsion-based adjuvant system containing 11.86 mg tocopherol [7]. The vaccines (0.5 ml per single dose) were administered intramuscularly into the deltoid region of the non-dominant arm for the single dose and into the deltoid region of each arm for the double dose.

### Study design and participants

The study took place between November 2006 and September 2009. The first part of this phase II, randomised, open-label study (NCT00397215) was conducted in multiple centres in Italy and Belgium and has been reported previously [13]. The long-term persistence of immune response and safety data at month 12 and month 24, reported here, were only evaluated for participants in Belgium, as specified in the amended study protocol; the cohort of subjects recruited in Italy was too small to warrant follow-up.

Male and female participants aged ≥61 years, who were in good health or who had well controlled underlying disease, were randomised (3:1:3:1) to one of four vaccine study groups to receive either two single doses of the AS03_A_-adjuvanted vaccine (1xH5N1-AS), two single doses of the non-adjuvanted vaccine (1xH5N1), two double doses of the AS03_A_-adjuvanted vaccine (2xH5N1-AS), or two double doses of the non-adjuvanted vaccine (2xH5N1). The vaccines were administered according to a two-dose schedule (day 0 and day 21). The majority of participants had not received an influenza vaccine for the 2006 to 2007 season and were vaccinated with *Fluarix™* Northern hemisphere 2006/2007 (GSK Vaccines) 3 weeks before administration of the H5N1 vaccine.

Participants in each group were stratified by age group (61 to 65, 66 to 70, and >70 years) to ensure equal enrolment in each age stratum. Treatment allocation at the investigator sites was performed using a central randomisation system.

The study was conducted in accordance with the Declaration of Helsinki and the International Conference on Harmonisation Good Clinical Practice guidelines. The protocol was approved by the independent ethics committee or institutional review board of each study centre (listed in Additional file 1). Written informed consent was obtained from each participant.

### Objectives

The primary objectives of this study were to evaluate the immunogenicity of the H5N1 vaccine 21 days after both the first and the second dose, as reported previously [13], and to assess persistence of the immune response for up to 2 years (reported in the current manuscript). Secondary objectives included safety assessment and evaluation of the cell-mediated immune response in terms of T helper 1-specific activation marker expression after *in vitro* re-stimulation of influenza-specific CD4^+^ and CD8^+^ T cells.

### Immunogenicity evaluation

We assessed the immunogenicity against two influenza strains: the vaccine-homologous A/Vietnam/1194/2004 strain and the vaccine-heterologous A/Indonesia/05/2005 strain.

Blood samples were taken at 12 and 24 months after the first vaccination. A haemagglutination inhibition (HI) assay was used to measure HI antibody titres in serially-diluted serum samples (initial dilution of 1:10, followed by two-fold serial dilution series using standard techniques [14]). The reported serum titre was the geometric mean of duplicate testing data. The assay cut-off was 10 (the reciprocal of the initial serum dilution); antibody titres below the cut-off were given an arbitrary value of half the cut-off for geometric mean titre (GMT) calculation. The following parameters were determined with 95% CIs at month 12 and month 24: H5N1 antibody GMTs, seroconversion rates (SCRs; defined as the percentage of participants with either a pre-vaccination titre <1:10 and a post-vaccination titre ≥1:40, or a pre-vaccination titre ≥1:10 and a ≥4-fold increase in post-vaccination titre) and seroprotection rate (SPR; defined as the percentage of participants with an HI titre ≥1:40).

The microneutralisation assay was performed as described previously [13]. Each serum sample was tested in triplicate. The 50% neutralisation titre of the serum was calculated according to the method of Reed and Muench [15]. The assay cut-off was 28; antibody titres below the cut-off were given an arbitrary value of half the cut-off for GMT calculation. Neutralising antibodies were evaluated in a subset of participants in the AS03_A_-adjuvanted vaccine groups at months 12 and 24 using the following parameters with their 95% CIs: H5N1 antibody GMTs and SCRs (defined as the percentage of vaccinees with a ≥4-fold increase in neutralising antibody titre at post-vaccination).

The cell-mediated immune response was assessed as described previously [13], and was only evaluated for the A/Vietnam/1194/2004 strain. Results were expressed as frequency of CD4^+^ or CD8^+^ T cells responding to the antigen and expressing at least two markers (CD40L, interferon-γ, interleukin-2 or tumour necrosis factor-α) per million CD4^+^ or CD8^+^ T cells in total. Response was measured at month 12 and month 24.

### Safety evaluation

We evaluated the occurrence of SAEs and AESIs (including autoimmune diseases and other immune-mediated inflammatory disorders) during the entire study. SAEs were defined as any medical occurrence that resulted in death, was a life-threatening event, resulted in disability or incapacity, or required hospitalisation or prolonged existing hospitalisation. Reactogenicity data have been reported previously [13].

### Statistical analysis

Determination of the target sample size has been described previously [13].

Analysis of humoral immunogenicity was performed on the month 24 according-to-protocol (ATP) persistence cohort, including all participants enrolled in Belgium who met all eligibility criteria, complied with protocol procedures, and had antibody assay results available. Cellular immunogenicity analyses were performed on the month 12 ATP persistence cohort for the month 12 timepoint and on the month 24 ATP persistence cohort for the month 24 timepoint. A subset of participants was randomly selected from each adjuvanted vaccine group for measurement of neutralising antibodies.

Analyses were performed using SAS 9.1 (SAS Institute Inc., Cary, NC, USA). Proc StatXact 5.0 (Cytel Inc., Cambridge, MA, USA) was used to calculate 95% CIs for GMT, SCR and SPR. The immunogenicity results for the follow-up study phases were reported descriptively.

## Results

**Figure 1:**
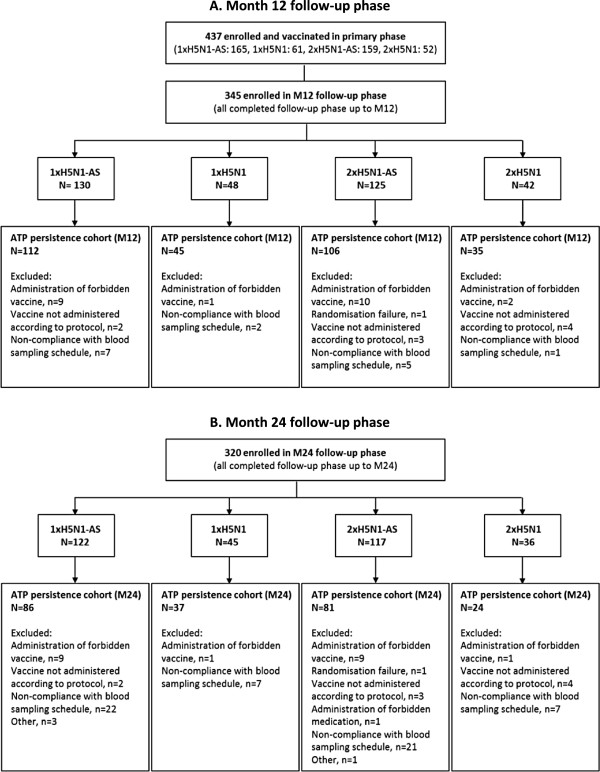
**Participant flow diagram.** Vaccination groups are: 1xH5N1-AS, single dose of the AS03_A_-adjuvanted vaccine; 1xH5N1, single dose of the non-adjuvanted vaccine; 2xH5N1-AS, double dose of the AS03_A_-adjuvanted vaccine; 2xH5N1, double dose of the non-adjuvanted vaccine. ATP, according-to-protocol; M, month; N, number of participants in the specified group; n, number of participants with the specified reason for elimination.

A total of 345 and 320 participants were enrolled in, and completed, the month 12 and month 24 follow-up phases of the study, respectively. The month 12 and month 24 ATP persistence cohorts included 298 and 228 participants, respectively (Figure 1A,B).

Demographic characteristics were similar across the study groups. In the ATP cohort for persistence at month 24, the overall mean age (± standard deviation) of participants at the time of first vaccination was 68.7 ± 6.18 years (range 61 to 89 years); 46.5% of participants were women, and the majority (95.2%) were of Caucasian/European heritage (Table 1).

**Table 1:** **Demographic characteristics of the participants (month 24 according-to-protocol persistence cohort)**

		1xH5N1-AS	1xH5N1	2xH5N1-AS	2xH5N1	Total
		N = 86	N = 37	N = 81	N = 24	N = 228
Age	Mean ± SD	68.5 ± 6.32	68.6 ± 5.15	69.2 ± 6.48	67.7 ± 6.30	68.7 ± 6.18
	Range (min–max)	61–85	61–81	61–89	61–85	61–89
Gender: n (%)	Female	42 (48.8)	15 (40.5)	39 (48.1)	10 (41.7)	106 (46.5)
Race: n (%)	White-Caucasian/European heritage	85 (98.8)	36 (97.3)	74 (91.4)	22 (91.7)	217 (95.2)
	White-Arabic/North African heritage	1 (1.2)	1 (2.7)	7 (8.6)	2 (8.3)	11 (4.8)

N, number of participants in the specified group; n (%), number of participants with the specified characteristic; SD, standard deviation. Vaccination groups are: 1xH5N1-AS, single dose of the AS03_A_-adjuvanted vaccine; 1xH5N1, single dose of the non-adjuvanted vaccine; 2xH5N1-AS, double dose of the AS03A-adjuvanted vaccine; 2xH5N1, double dose of the non-adjuvanted vaccine.

### Haemagglutination inhibition antibody response

**Figure 2:**
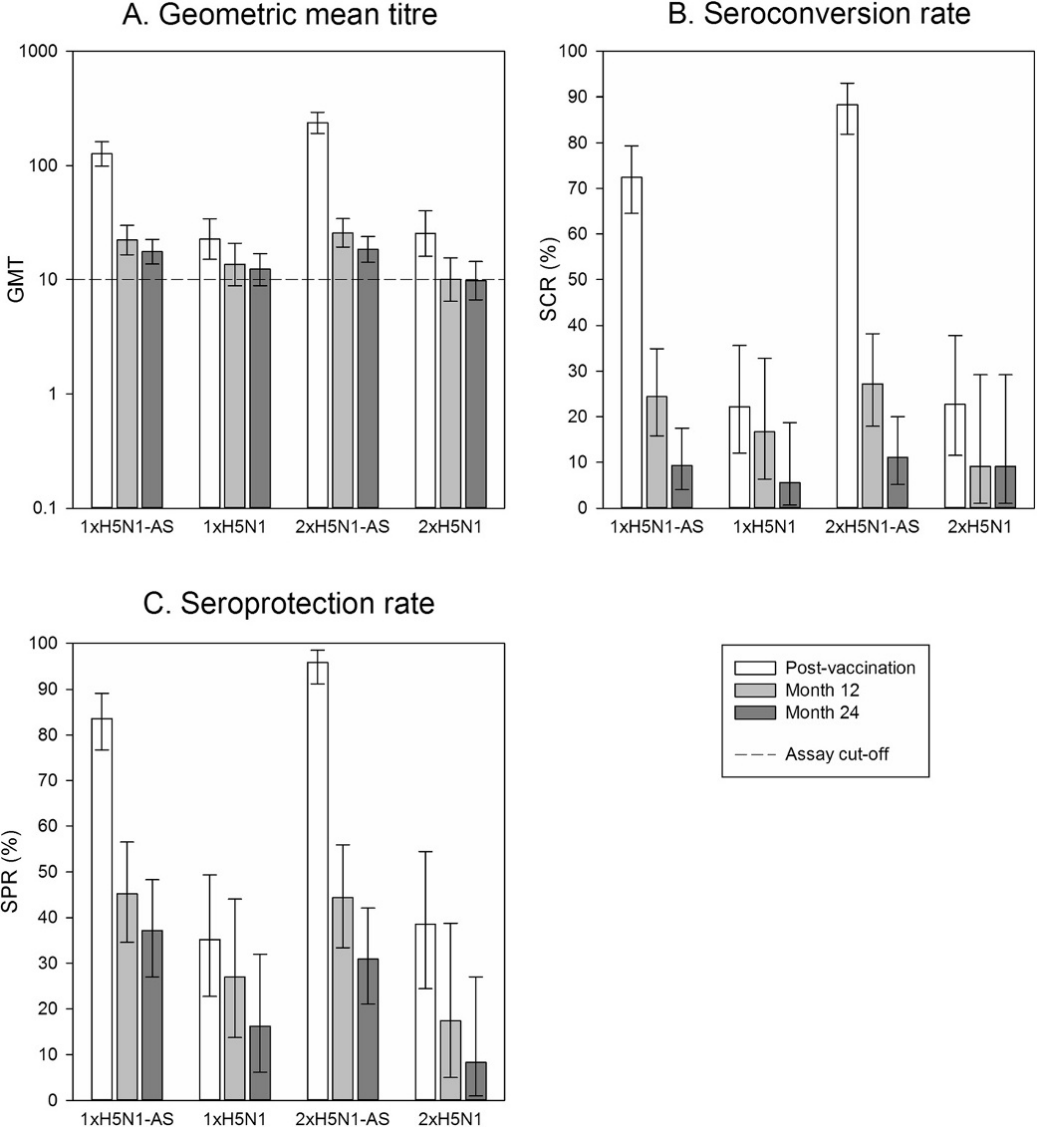
**H5N1 haemagglutination inhibition antibody response against A/Vietnam/1194/2004.** Vaccination groups are: 1xH5N1-AS, single dose of the AS03_A_-adjuvanted vaccine; 1xH5N1, single dose of the non-adjuvanted vaccine; 2xH5N1-AS, double dose of the AS03_A_-adjuvanted vaccine; 2xH5N1, double dose of the non-adjuvanted vaccine. Seroconversion rate (SCR) was defined as the percentage of participants with either a pre-vaccination titre <1:10 and a post-vaccination titre ≥1:40, or a pre-vaccination titre ≥1:10 and a ≥4-fold increase in post-vaccination titre; and seroprotection rate (SPR) was defined as the percentage of participants with a haemagglutination inhibition titre ≥1:40. GMT, geometric mean titre. Error bars indicate the 95% confidence intervals. Post-vaccination = day 42 (data also shown in [13]). Post-vaccination results are shown for the according-to-protocol immunogenicity cohort (1xH5N1-AS: N =152, 1xH5N1: N =54, 2xH5N1-AS: N =145, 2xH5N1: N = 44); month 12 and month 24 results for the month 24 according-to-protocol persistence cohort (1xH5N1-AS: N = 86, 1xH5N1: N = 37, 2xH5N1-AS: N =81, 2xH5N1: N = 24); N = maximum number of participants with available results.

**Figure 3:**
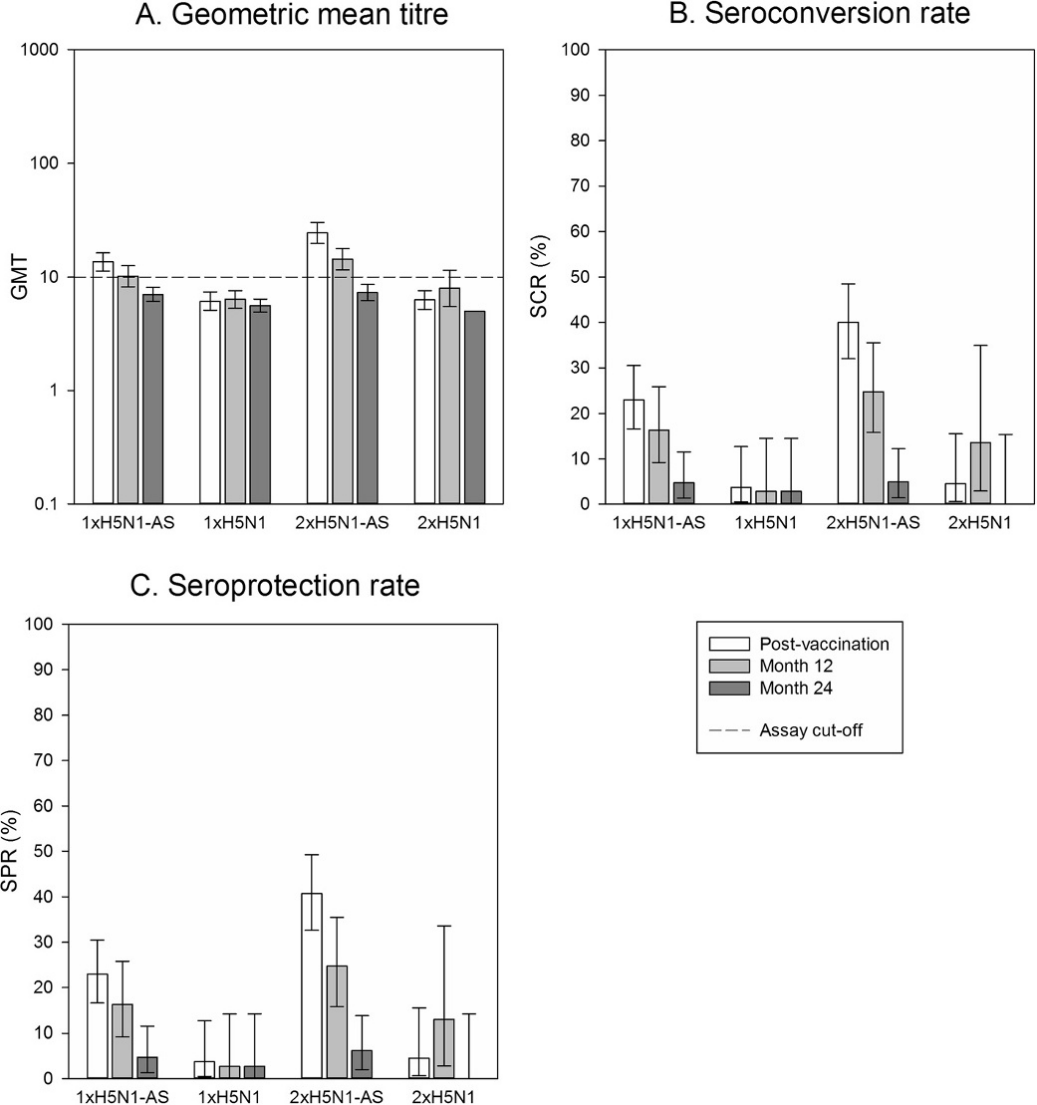
**H5N1 haemagglutination inhibition antibody response against the heterologous A/Indonesia/05/2005 strain.** Vaccination groups are: 1xH5N1-AS, single dose of the AS03_A_-adjuvanted vaccine; 1xH5N1, single dose of the non-adjuvanted vaccine; 2xH5N1-AS, double dose of the AS03_A_-adjuvanted vaccine; 2xH5N1, double dose of the non-adjuvanted vaccine. Seroconversion rate (SCR) was defined as the percentage of participants with either a pre-vaccination titre <1:10 and a post-vaccination titre ≥1:40, or a pre-vaccination titre ≥1:10 and a ≥4-fold increase in post-vaccination titre; and seroprotection rate (SPR) was defined as the percentage of participants with a haemagglutination inhibition titre ≥1:40. GMT, geometric mean titre. Error bars indicate the 95% confidence intervals. Post-vaccination = day 42. Post-vaccination results are shown for the according-to-protocol immunogenicity cohort (1xH5N1-AS: N =152, 1xH5N1: N = 54, 2xH5N1-AS: N = 145, 2xH5N1: N = 44); month 12 and month 24 results for the month 24 according-to-protocol persistence cohort (1xH5N1-AS: N = 86, 1xH5N1: N = 37, 2xH5N1-AS: N = 81, 2xH5N1: N =24); N = maximum number of participants with available results.

At month 12, the percentages of participants who remained seropositive for HI antibodies against A/Vietnam/1194/2004 were 62.8% (95% CI 51.7% to 73.0%) and 77.8% (95% CI 67.2% to 86.3%) in the adjuvanted 1xH5N1-AS and 2xH5N1-AS groups, respectively, compared to 48.6% (95% CI 31.9% to 65.6%) and 39.1% (95% CI 19.7% to 61.5%) in the non-adjuvanted 1xH5N1 and 2xH5N1 groups, respectively. At month 24, seropositivity rates were in a similar range to those at month 12; 62.8% (95% CI 51.7% to 73.0%) in the 1xH5N1-AS group, 70.4% (95% CI 59.2% to 80.0%) in the 2xH5N1-AS group, 54.1% (95% CI 36.9% to 70.5%) in the non-adjuvanted 1xH5N1 group, and 45.8% (95% CI 25.6% to 67.2%) in the non-adjuvanted 2xH5N1 group.GMTs for HI antibodies against A/Vietnam/1194/2004 waned considerably as time elapsed after the last primary dose. At month 12, HI antibody GMTs were 22.2 (95% CI 16.5 to 29.9) in the 1xH5N1-AS group, 25.7 (95% CI 19.3 to 34.3) in the 2xH5N1-AS group, 13.6 (95% CI 8.9 to 20.8) in the 1xH5N1 group and 10.0 (95% CI 6.5 to 15.5) in the 2xH5N1 group (Figure 2A). At month 24, GMTs were 17.6 (95% CI 13.7 to 22.5) for 1xH5N1-AS, 18.4 (95% CI 14.2 to 23.8) for 2xH5N1-AS, 12.3 (95% CI 8.9 to 16.9) for 1xH5N1 and 9.8 (95% CI 6.7 to 14.4) for 2xH5N1 (Figure 2A). In comparison, pre-vaccination GMTs were 11.5 (95% CI 8.7 to 15.1) for 1xH5N1-AS, 9.7 (95% CI 7.4 to 12.5) for 2xH5N1-AS, 9.3 (95% CI 6.3 to 13.6) for 1xH5N1 and 6.8 (95% CI 5.4 to 8.7) for 2xH5N1.For SCR and SPR, CHMP criteria were no longer fulfilled at both month 12 and month 24 for the HI immune response against any of the two strains tested (Figure 2B,C and Figure 3B,C). At month 24, HI antibody seroprotection rates were 37.2% (95% CI 27.0% to 48.3%) in the 1xH5N1-AS group, 30.9% (95% CI 21.1% to 42.1%) in the 2xH5N1-AS group, 16.2% (95% CI 6.2% to 32.0%) in the non-adjuvanted 1xH5N1 group, and 8.3% (95% CI 1.0% to 27.0%) in the non-adjuvanted 2xH5N1 group.The heterologous HI immune response against the A/Indonesia/05/2005 strain was lower than against the A/Vietnam/1194/2004 strain in all groups (Figure 3A-C), with a GMT of 7.0 (95% CI 6.1 to 8.1) for 1xH5N1-AS, 7.3 (95% CI 6.2 to 8.6) for 2xH5N1-AS, 5.6 (95% CI 4.9 to 6.4) for 1xH5N1 and 5.0 (95% CI 5.0 to 5.0) for 2xH5N1 at month 24; these values were below the cut-off of 10. GMTs remained higher than baseline for the adjuvanted groups while those of the non-adjuvanted groups were in the same range as baseline values; pre-vaccination GMTs were 5.1 (95% CI 5.0 to 5.2) for 1xH5N1-AS, 5.1 (95% CI 5.0 to 5.3) for 2xH5N1-AS, 5.1 (95% CI 4.9 to 5.3) for 1xH5N1, and 5.0 (95% CI 5.0 to 5.0) for 2xH5N1.

### Neutralising antibody response

**Figure 4:**
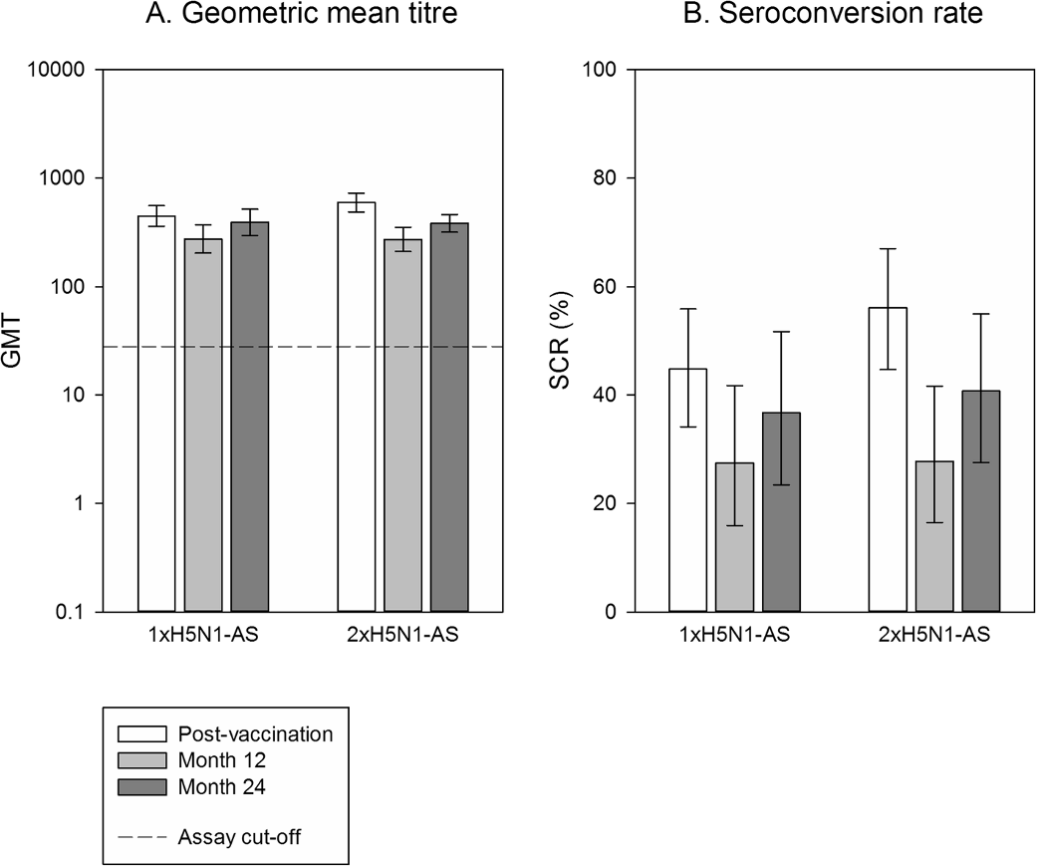
**Neutralising antibody response against A/Vietnam/1194/2004 in the AS03**_**A**_ **-adjuvanted groups.** Vaccination groups are: 1xH5N1-AS, single dose of the AS03_A_-adjuvanted vaccine; 1xH5N1, single dose of the non-adjuvanted vaccine; 2xH5N1-AS, double dose of the AS03_A_-adjuvanted vaccine; 2xH5N1, double dose of the non-adjuvanted vaccine. Seroconversion rate (SCR) was defined as the percentage of vaccinees with a ≥4-fold increase in neutralising antibody titre at post-vaccination. GMT, geometric mean titre. Error bars indicate the 95% confidence intervals. Post-vaccination = day 42 (data also shown in [13]). Post-vaccination results are shown for a subset of the according-to-protocol immunogenicity cohort (1xH5N1-AS: N =87, 2xH5N1-AS: N =82), month 12 and month 24 results for a subset of the month 24 according-to-protocol persistence cohort (1xH5N1-AS: N =51, 2xH5N1-AS: N =54); N = maximum number of participants with available results.

**Figure 5:**
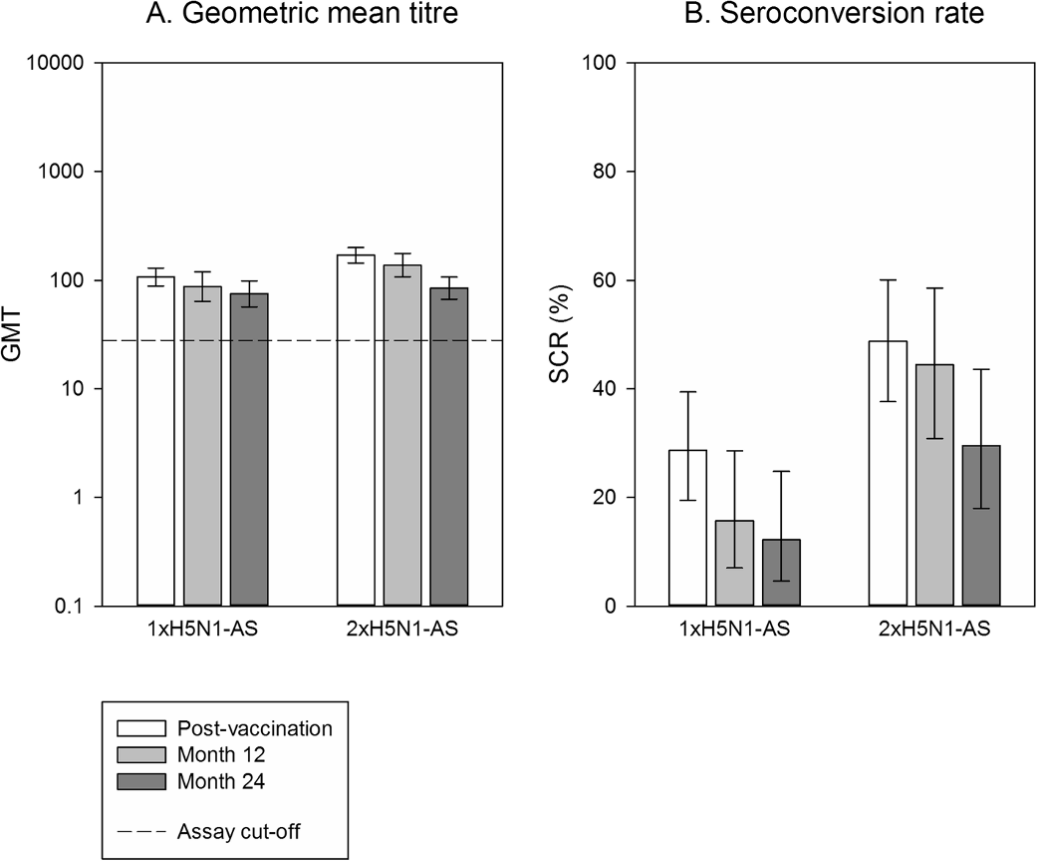
**Neutralising antibody response against the heterologous A/Indonesia/05/2005 strain in the AS03**_**A**_ **-adjuvanted groups.** Vaccination groups are: 1xH5N1-AS, single dose of the AS03_A_-adjuvanted vaccine; 1xH5N1, single dose of the non-adjuvanted vaccine; 2xH5N1-AS, double dose of the AS03_A_-adjuvanted vaccine; 2xH5N1, double dose of the non-adjuvanted vaccine. Seroconversion rate (SCR) was defined as the percentage of vaccinees with a ≥4-fold increase in neutralising antibody titre at post-vaccination. GMT, geometric mean titre. Error bars indicate the 95% confidence intervals. Post-vaccination = day 42. Post-vaccination results are shown for a subset of the according-to-protocol immunogenicity cohort (1xH5N1-AS: N =87, 2xH5N1-AS: N =82), month 12 and month 24 results for a subset of the month 24 according-to-protocol persistence cohort (1xH5N1-AS: N =51, 2xH5N1-AS: N =54); N = maximum number of participants with available results.

All participants in the adjuvanted groups remained seropositive for neutralising antibodies against A/Vietnam/1194/2004 at both month 12 and month 24, with a decrease in antibody GMTs and SCRs compared to 3 weeks post-vaccination (Figure 4A,B). The antibody GMTs remained higher than the pre-vaccination values, which were 131.8 (95% CI 95.4 to 182.1) for the 1xH5N1-AS group and 115.6 (95% CI 90.0 to 148.4) for the 2xH5N1-AS group.Most participants (≥84.1%) remained seropositive for neutralising antibodies against the heterologous A/Indonesia/05/2005 strain, with a decrease in antibody GMTs at both month 12 and month 24 (Figure 5). Pre-vaccination GMTs were 50.4 (95% CI 38.6 to 65.8) for 1xH5N1-AS and 36.6 (95% CI 27.8 to 48.1) for 2xH5N1-AS.

### Cell-mediated immunity

**Figure 6:**
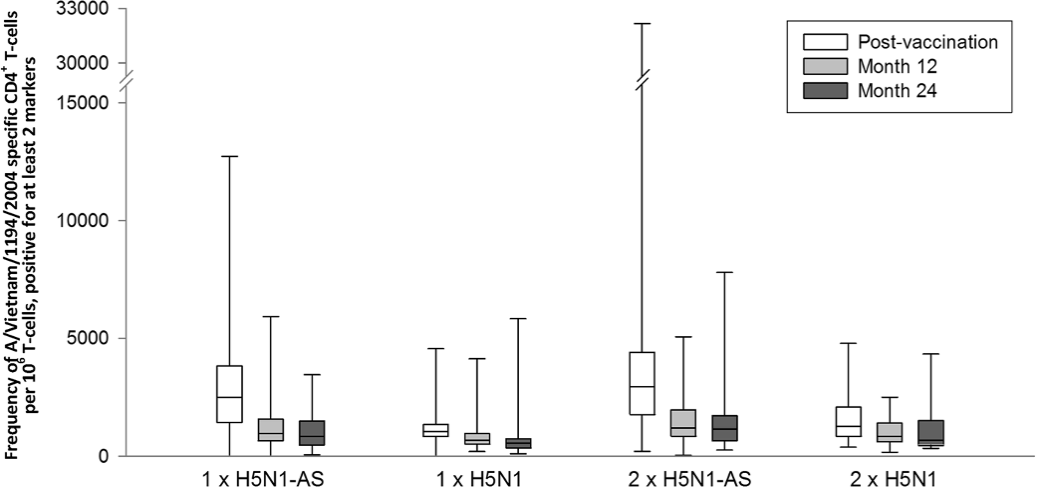
**CD4** ^**+**^ **T cells specific for A/Vietnam/1194/2004 split-virus antigen, positive for ≥2 immunological markers.** Antigen-specific CD4^+^ T cells expressing ≥2 of the following immunological markers: CD40L, interleukin-2, tumour necrosis factor alpha, or interferon-gamma. Line inside the box, median value; top line of box, third quartile; bottom line of box, first quartile; lower limit of error bar, minimum value; upper limit of error bar, maximum value. Vaccination groups are: 1xH5N1-AS, single dose of the AS03_A_-adjuvanted vaccine; 1xH5N1, single dose of the non-adjuvanted vaccine; 2xH5N1-AS, double dose of the AS03_A_-adjuvanted vaccine; 2xH5N1, double dose of the non-adjuvanted vaccine. Post-vaccination = day 42 (data also shown in [13]). Post-vaccination results are shown for the according-to-protocol immunogenicity cohort (1xH5N1-AS: N =120, 1xH5N1: N =43, 2xH5N1-AS: N =118, 2xH5N1: N =30), month 12 results for the month 12 according-to-protocol persistence cohort (1xH5N1-AS: N =90, 1xH5N1: N =41, 2xH5N1-AS: N =91, 2xH5N1: N =30) and month 24 results for the month 24 according-to-protocol persistence cohort (1xH5N1-AS: N =73, 1xH5N1: N =34, 2xH5N1-AS: N =72, 2xH5N1: N =22); N = number of participants with available results.

Antigen-specific CD4^+^ T-cell responses decreased between day 42 and month 12, and remained within a similar range at month 24. At month 24, the median frequency of antigen-specific CD4^+^ T cells per 10^6^ T cells (25th quartile; 75th quartile) was 852 (482; 1477) for 1xH5N1-AS, 1147 (662; 1698) for 2xH5N1-AS, 556 (343; 749) for 1xH5N1 and 673 (465; 1497) for 2xH5N1 (Figure 6). No CD8^+^ T-cell responses were observed.

### Safety

Between months 6 and 12, 12 non-fatal SAEs were reported by 9 of 345 participants (2 in the 2xH5N1 group, 3 in the 2xH5N1-AS group and 4 in the 1xH5N1-AS group). One SAE was considered causally related to vaccination by the investigator (right inferior lobar pneumonia of undetermined cause, with onset 299 days after the second dose in the 2xH5N1 group).

Between months 12 and 24, 29 non-fatal SAEs were reported by 25 of 320 participants (2 in the 2xH5N1 group, 9 in the 1xH5N1-AS group and 7 each in the 1xH5N1 and the 2xH5N1-AS group). None of these SAEs were considered to be causally related to vaccination.

No AESIs were reported between months 6 and 24.

## Discussion

It is well established that the immune response to vaccination is weaker in older individuals [12]. We previously reported that two injections of a double dose of the AS03_A_-adjuvanted H5N1 pandemic vaccine induced a stronger immune response than two injections of a single dose in participants (>60 years old), although administration of two single vaccine doses was sufficient to meet the licensing criteria [13]. In addition, we reported that HI and neutralizing antibodies persisted at 6 months after vaccination with the AS03_A_-adjuvanted H5N1 pandemic vaccine formulations [13].

Here, we showed that persistence of the antibodies was observed up to 2 years after vaccination, although expectedly antibody GMTs decreased considerably over time. Two double-dose vaccinations had no effect on the persistence of the immune response compared to two single-dose injections. This, together with our previous finding that the single-dose regimen was sufficient to meet licensing criteria, further supports the use of the registered adult dose and schedule in the elderly population. This knowledge is valuable in terms of simplification of the vaccination schedule across adulthood with no need for a specific dosage for elderly individuals, and it reduces the strain on the limited global production capacity of influenza antigen in case of a pandemic. A similar pattern in adults and elderly (≥61 years) has also been described for two doses of an MF59-adjuvanted H5N1 pre-pandemic vaccine [16]. Two doses of another AS03_A_-adjuvanted H5N1 vaccine were also sufficient to meet licensing criteria in older persons (≥65 years), although GMTs were lower than in younger adults; persistence of the antibody response was observed up to 182 days after the first dose in both age groups [17].

Higher HI antibody levels were observed in the adjuvanted vaccine groups than in the non-adjuvanted groups up to month 24, although the levels in all groups were low. Addition of the AS03_A_ adjuvant thus appears to improve persistence of the immune response.

The observed neutralizing antibody titres for the vaccine homologous strain tended to be higher at month 24 than at month 12. However, this may most likely be explained by variability between assay runs, as the overall GMT values were low.

The HI immune response against the heterologous A/Indonesia/05/2005 strain was lower than against the A/Vietnam/1194/2004 strain in all groups, and GMTs were below the assay cut-off of 10 except for the 2xH5N1-AS group at month 12. Neutralizing antibody GMTs against A/Indonesia/05/2005 decreased compared to the post-vaccination timepoint, but remained well above the assay cut-off and also tended to be higher than the pre-vaccination GMTs (although the 95% CIs were overlapping for the 1xH5N1-AS group).

The use of antibody titres as a sole indicator for influenza vaccine efficacy is of limited value in older people; indeed, available data suggest that the cell-mediated immune response should also be taken into account in the evaluation of vaccine efficacy in this population [18–21]. T helper 1-cell frequencies have been shown to be a good early predictor of seroprotection after booster vaccination [22]. Similarly, we previously observed higher CD4^+^ T-cell responses against the A/Vietnam/1194/2004 strain for the adjuvanted than non-adjuvanted groups at day 21, which corresponded to higher post-dose two seroprotection rates in the adjuvanted groups [13]. Our current results indicated similar patterns of persistence of HI antibodies, neutralising antibodies, and CD4^+^ T-cell responses, with a trend for improved persistence of the cell-mediated immune response in the adjuvanted vaccine groups. No CD8^+^ T-cell responses were observed, in line with our previous observations [13].

No assessment of memory B-cell response was performed in the current study. However, in a long-term booster study with AS03_A_-adjuvanted H5N1 vaccine performed in a younger adult population from Asia, the significant responses observed after one dose of heterologous booster vaccine up to 3 years after priming allow to infer that a memory response is induced [23].

## Conclusions

During the 2-year follow-up period, antibody levels declined substantially in all groups. Higher GMTs for HI antibodies were observed in the adjuvanted vaccine groups compared to the non-adjuvanted vaccine groups. The CD4^+^ T-cell responses also appeared higher in the adjuvanted groups at both month 12 and month 24. This study, together with the results from the preceding study, therefore supports the suitability of the adult dosage and schedule of the AS03_A_-adjuvanted H5N1 pandemic vaccine for elderly persons.

No safety concern was observed up to 24 months after primary vaccination.

Prepandrix and Fluarix are trademarks of the GlaxoSmithKline group of companies.

## Electronic supplementary material

Additional file 1: **Independent Ethics Committees/Institutional Review Boards that approved the study.** (DOCX 14 KB)

## Abbreviations

AESI: adverse events of specific interest
ATP: according-to-protocol
CHMP: Committee for Human Medicinal Products
GMT: geometric mean titre
HI: haemagglutination inhibition
SAE: serious adverse event
SCR: seroconversion rate
SPR: seroprotection rate.
