# Supplementary material for: Long-term outcome of the humoral and cellular immune response of an H5N1 adjuvanted influenza vaccine in elderly persons: 2-year follow-up of a randomised open-label study
Source: Trials. 2014 Oct 29;15:419. doi: 10.1186/1745-6215-15-419 (PMC4233031; doi:10.1186/1745-6215-15-419)
Supplement: Supplementary file 1 — Additional file 1: Independent Ethics Committees/Institutional Review Boards that approved the study. (DOCX 14 KB) [file 13063_2013_2294_MOESM1_ESM.docx]

**Supplement 1. Independent Ethics Committees / Institutional Review Boards that approved the study**

Ethics Committee Erasme Hospital
808, Route de Lennik,

1070 Brussels, Belgium

Comitato Etico
Azienda Ospedaliera Universitaria S.Martino

Largo Rosanna Benzi, 10

16132 Genova, Italy

Comitato Etico
c/o Ufficio Ricerche Cliniche
Fondazione Centro S.Raffaele del Monte Tabor
Via Olgettina, 60
20132 Milano, Italy

Bioethics Committee
Piazza Igea Ragusa
97100 Ragusa, Italy

Bioethics Committee
Via Monte Grappa, 82

07100 Sassari, Italy

Comitato Etico
Policlinico Universitario
Via S.Giorgio, 12
09100 Cagliari, Italy
